# Ozone decomposition

**DOI:** 10.2478/intox-2014-0008

**Published:** 2014-11-15

**Authors:** Todor Batakliev, Vladimir Georgiev, Metody Anachkov, Slavcho Rakovsky, Gennadi E. Zaikov

**Affiliations:** 1 Institute of Catalysis, Bulgarian Academy of Sciences, Sofia, Bulgaria; 2 N.M. Emanuel Institute of Biochemical Physics, Russian Academy of Sciences, Moscow, Russia; * This join work is dedicated to the 80^th^ Anniversary of Prof. Gennadi Zaikov

**Keywords:** ozone, catalysts, decomposition, synthesis, kinetics, mechanism

## Abstract

Catalytic ozone decomposition is of great significance because ozone is a toxic substance commonly found or generated in human environments (aircraft cabins, offices with photocopiers, laser printers, sterilizers). Considerable work has been done on ozone decomposition reported in the literature. This review provides a comprehensive summary of the literature, concentrating on analysis of the physico-chemical properties, synthesis and catalytic decomposition of ozone. This is supplemented by a review on kinetics and catalyst characterization which ties together the previously reported results. Noble metals and oxides of transition metals have been found to be the most active substances for ozone decomposition. The high price of precious metals stimulated the use of metal oxide catalysts and particularly the catalysts based on manganese oxide. It has been determined that the kinetics of ozone decomposition is of first order importance. A mechanism of the reaction of catalytic ozone decomposition is discussed, based on detailed spectroscopic investigations of the catalytic surface, showing the existence of peroxide and superoxide surface intermediates.

## 1. Introduction

In recent years, scientific research in all leading countries of the world has been aimed primarily at solving the deep environmental problems on the planet, including air pollution and global warming. One of the factors affecting negatively these processes is the presence of ozone in ground atmospheric layers. This is a result of the wide use of ozone in many important industrial processes, such as cleaning of potable water and soil, disinfection of plant and animal products, textile bleaching, complete oxidation of waste gases from the production of various organic chemicals, sterilization of medical supplies, *etc.* (Rakovsky *et al.*, 2007).

The history of ozone chemistry as a research field began immediately after its discovery by Schönbein in 1840 (Schönbein, 1840). The atmospheric ozone is found mainly in the so-called “ozone layer” at a height of 15 to 30 km above the earth surface wherein the ozone concentration ranges from 1 to 10 ppm (Wiley, 1991). The ozone synthesis at that altitude runs photochemically through the influence of solar radiation on molecular oxygen. The atmospheric ozone is invaluable to all living organisms because it absorbs the harmful ultraviolet radiation from the sun. The study of the kinetics and mechanism of ozone reactions in modern science is closely related to solving the ozone holes problem, reflecting the trend of recent decades to the depletion of the atmospheric ozone layer.

Ozone has oxidation, antibacteriological and antiviral properties that make it widely used in the treatment of natural, industrial and polluted waters, swimming pools, contaminated gases, for medical use, *etc.* Catalytic decomposition of residual ozone is imperative because from environmental point of view the release of ozone in the lower atmosphere has negative consequences.

The presence of ozone in the surrounding human environment (airplane cabins, copiers, laser printers, sterilizers, *etc.*) also raises the issue of its catalytic decomposition as ozone is highly toxic above concentrations of 0.1 mg.m^−3^ (Razumovskii *et al.*, 1983; Brown *et al.*, 2002) and can damage human health. The use of catalysts based on transition metal oxides is emerging as very effective from an environmental perspective, providing at the same time a practical and inexpensive method for the decomposition of residual ozone.

## 2. Some physico-chemical properties of ozone

Ozone is an allotropic modification of oxygen that can exist in all three physical conditions. In normal conditions, ozone is a colorless gas with a pungent odor, while at very low concentrations (up to 0.04 ppm) it can give the feeling of pleasant freshness. A characteristic property of ozone odor is the fact that it is easily addictive and at the same time hazardous for people who work with ozone in view of its high toxicity at concentrations above the limit (>0.1 mg.m^−3^).

**Figure 1:**
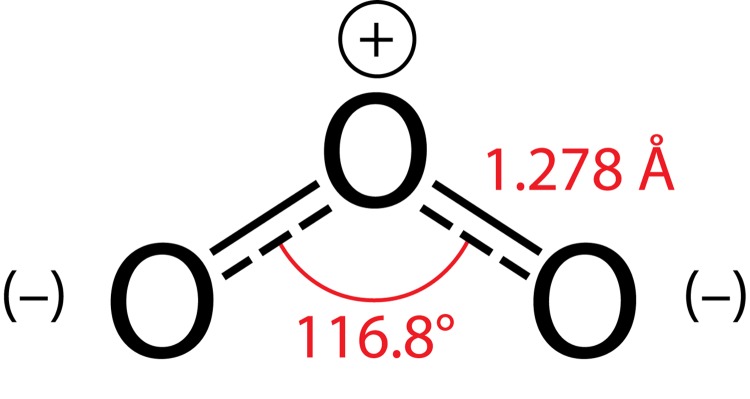
Structure of O_3_ molecule.

When the ozone concentration exceeds 15–20% it has a blue color. At atmospheric pressure and temperature of 161.3 K, the ozone becomes fluid and is of deep blue color. It cures at 80.6 K by acquiring a dark purple color (Lunin *et al.*, 1998). Ozone is explosive in all three physical conditions. Work with ozone concentrations of 0 to 15% can be considered safe (Razumovskii *et al.*, 1983). The danger of ozone explosion is a function of its thermodynamic instability (ΔG°298=–163 kJ.mol^−1^) and ozone decomposition to diatomic oxygen is a thermodynamically favorable process with heat of reaction ΔH°298=–138 kJ.mol^−1^ (Perry & Green, 1997). The ozone molecule consists of three oxygen atoms located at the vertices of an obtuse-angled triangle with a central angle of 116°8’±5’ and length of O–O-bond: ρ_o–o_ = 0.1278±0.0003 nm (Figure 1).

In gas phase, the ozone molecule is a singlet biradical while in liquid phase it reacts generally as a dipolar ion. The homogeneous reaction of pure gaseous ozone decomposition is characterized by a certain velocity at sufficiently high temperatures. The kinetics study of ozone thermal decomposition is also complicated by the fact that above a certain critical temperature the steady kinetic decomposition is transformed into explosion, subsequently passing to detonation (Rakovsky *et al.*, 2007). According to Thorp (1955), ozone detonation is observed above 105 °C. The gaseous ozone is characterized by different times of half-life, depending on the temperature (Table 1).

**Table 1:** Half-life of ozone.

Temp. (°C)	Half-life
−50	3-months
−35	18-days
−25	8-days
20	3-days
120	1.5-hours
250	1.5-seconds

**Figure 2:**
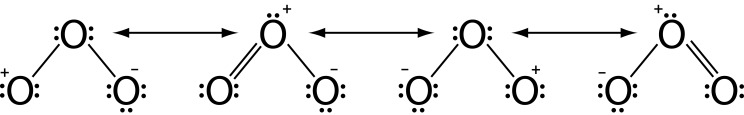
Resonance structures of ozone.

The ozone structure is resonance stabilized, which is one of the reasons for its resistance against decomposition at low temperatures (Figure 2).

In most reactions with inorganic compounds, ozone reacts with participation of one atom oxygen and the other two are separate as O_2_. Typically, the elements are oxidized to their highest oxidation states. For example, manganese is oxidized to [MnO]^4−^, halogen oxides to metals (ClO_2_, Br_2_O_5_), ammonia to NH_4_NO_3_, nitrogen oxides pass into N_2_O_5_ (Kutsuna *et al.*, 1994; Naydenov *et al.*, 1995; Rakitskaya *et al.*, 1994).

Ozone has spectral characteristics from the IR-region to the vacuum UV-region, which are present in a significant number of works as the majority of them are carried out with gaseous ozone (Tanaka *et al.*, 1953; De More & Paper, 1964; Beitker & Schurath, 1974; Inn & Tanaka, 1953; Griggs, 1968; Taube, 1957; Galimova *et al.*, 1973). It should be noted that most researchers who use the spectrophotometric method for analysis of ozone work in the main area of the spectrum 200÷310 nm, where is the wide band with a maximum at ∼255 nm for gaseous ozone (De More & Paper, 1964; Inn & Tanaka, 1953; Griggs, 1968; Taube, 1957). That maximum is characterized with a high value of the coefficient of extinction (Table 2).

**Table 2:** Coefficient of extinction (e, L.mol^−1^.cm^−1^) of gaseous ozone in UV-region (Alexandrov *et al*., 1983)

l, nm	(Kondratev, 1990)	(Vupputuri, 1988)	(Heisig *et al.,* 1997)	(Emelyanova *et al.,* 1965)
253.6*	2981	3024	2952	3316
270	–	–	–	2302
289.4	383	337.5	387.5	–
296.7**	150.5	153.4	156.8	–
302.2	74.4	–	74.4	–
334.2	1.50	–	1.46	–

* The values of e are 1830 L.mol^−1^.cm^−1^ at 254.0 nm (Razumovskii *et al.*, 1983) and 3020 L.mol^−1^.cm^−1^ at 253.6 nm (Beitker & Schurath, 1974)

** e = 160 L.mol^−1^.cm^−1^ at 295 nm (Galimova *et al.*, 1973)

## 3. Ozone synthesis and analysis

Ozone for industrial aims is synthesized from pure oxygen by thermal, photochemical, chemical, electrochemical methods, in all forms of electrical discharge and under the action of a particle stream (Rakovsky *et al.*, 2007). The synthesis of ozone is carried out by the following reactions shown in the scheme:

1 $${\text{O}}_{2}+({\text{e}}^{-},\text{hv},\text{T})\to 2\text{O}({\text{O}}_{2}^{*})$$

2 $${\text{O}}_{2}+\text{O}+\text{M}\to {\text{O}}_{3}+\text{M}$$

3 $${\text{O}}_{2}^{*}+{\text{O}}_{2}\to {\text{O}}_{3}+\text{O}$$

where: M is every third particle.

At low temperatures, the gas consists essentially of molecular oxygen and at higher by atomic oxygen. There is no area of temperatures at normal pressure wherein the partial pressure of ozone is significant. The maximum steady-state pressure which is observed at the temperature of 350 K is only 9.10^−7^ bar. The values of the equilibrium constant of reaction (2) at different temperatures are presented in Table 3 (Hon & Yan, 1993).

**Table 3:** Equilibrium constant (K_e_) of reaction (2) depending on the temperature.

T, K	1500	2000	3000	4000	5000	6000
K_e_, M	1 662.10^−11^	4 413.10^−7^	1 264.10^−2^	2.104	48.37	382.9

At high temperatures, when the concentration of atomic oxygen is high, the equilibrium of reaction (2) is moved to the left and the ozone concentration is low. At low temperatures, the equilibrium is shifted to the right, but through the low concentration of atomic oxygen the ozone content is negligible. For synthesis of significant concentrations of ozone it is necessary to combine two following conditions: 1) low temperature and 2) the formation of superequilibrium concentrations of atomic oxygen. It is possible to synthesize superequilibrium atomic concentrations of oxygen at low temperatures by using non-thermal processes, such as dissociation of oxygen with particle flow, electrons, hν, electrochemical and chemical influences. At low temperatures, ozone will always be formed when there is a process of oxygen dissociation. Higher concentrations of ozone may be obtained by thermal methods providing storage (“quenching”) of superequilibrium concentrations of atomic oxygen at low temperatures. Photochemical synthesis of ozone occurs upon irradiation of gaseous or liquid oxygen by UV radiation with the wavelength λ<210 nm (Deninno & McCarthy, 1997). It is assumed, that the formation of ozone in the presence of radiation with a wavelength in the range of 175<λ<210 to be associated with the formation of excited oxygen molecules (Claudia *et al.*, 1994):

4 $${\text{O}}_{2}+\text{hv}\to ({\text{O}}_{2}^{*})$$

5 $${\text{O}}_{2}^{*}+{\text{O}}_{2}\to {\text{O}}_{3}+\text{O}$$

6 $${\text{O}}_{2}+\text{O}+\text{M}\to {\text{O}}_{3}+\text{M}$$

The photochemical generation of ozone has an important role in atmospheric processes, yet it is hard to apply for industrial application because of the high energy costs (32 kWh.kg^−1^ ozone) for the preparation of high-energy shortwave radiation. Nowadays the industrial synthesis of ozone happens mainly by any of the electric discharge methods by passing oxygen containing gas through high-voltage (8–10 kV) electrodes. The aim is creation of conditions in which oxygen is dissociated into atoms. This is possible in all forms of electrical discharges: smoldering, silent, crown, arc, barrier, *etc.* The main reason for the oxygen dissociation is the hit of molecular oxygen with electrons accelerated in the electric field (Rakovsky *et al.*, 2007; Lunin *et al.*, 1998), when part of the kinetic energy is transformed in energy of dissociation of the bond O–O and excitation of oxygen molecules. In general, molecule oxygen dissociates in two oxygen atoms, then in the final stage two molecules of oxygen form one oxygen atom and one ozone molecule:

7 $${\text{O}}_{2}+\text{e}\to 2\text{O}\;({\text{O}}_{2}^{*})$$

8 $${\text{O}}_{2}+\text{O}+\text{M}\to {\text{O}}_{3}+\text{M}$$

9 $${\text{O}}_{2}^{*}+{\text{O}}_{2}\to {\text{O}}_{3}+\text{O}$$

**Figure 3:**
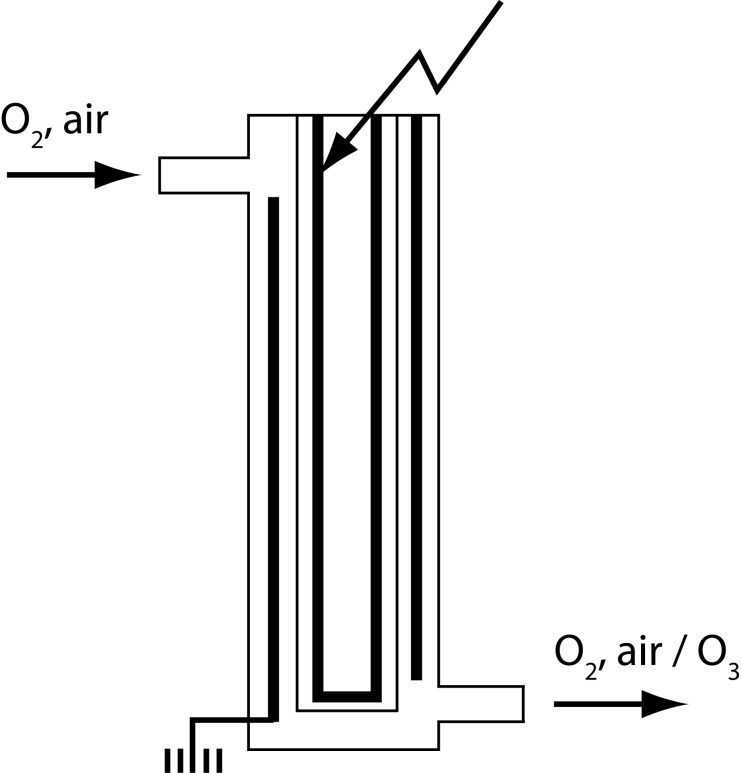
Principal scheme of Siemens ozonator.

Technically, the synthesis of ozone is carried out in discharge tubes – ozonators. One of the most commonly used type of tubes are Siemens ozonators (Figure 3).

This ozonator represents two coaxical pipes brazed one to another, supported inside of the inner side and outside of the outer side with electrically conductive coating of aluminum, silver, copper and the like. It is submitted to high voltage up to 20 kV and dry and pure oxygen is left to pass through the ozonator. The ozone is synthesized on the other end of the ozonator. Ozone concentration depends on the parameters of the current – voltage, frequency and power properties of the ozonator – thickness, length and type of glass tube, the distance between the electrodes and temperature. The ozone concentration depends on the current parameters – voltage, frequency and power, on the properties of the ozonator – thickness, length and type of glass tube, on the distance between the electrodes, and also on the temperature.

The analysis of ozone concentration is developed by various physical and chemical methods discussed in detail in the monograph (Razumovskii & Zaikov, 1974). It should be noted that preference is given to the spectrophotometric method compared with the iodometric method, determined by the lack of need of continuous pH monitoring during ozone analysis and also by the possibility of direct observation of the inlet and outlet ozone concentrations in the system, allowing precise fixing of the experimental time and its parameters. For determination of the ozone amount not absolute values of concentration but rather ratios of the proportional values of optical densities can be used, excluding the error influence at expense of the inaccuracy in calibration of the instrument.

## 4. Ozone decomposition

The reaction of ozone on the surface of solids is of interest from different points of view. Along with the known gas cycles depleting the atmospheric ozone, a definitive role is played also by its decomposition on aerosols, so that quantity is growing at the expense of the anthropogenic factor. The growth of ozone used in chemical industries set up the task for decomposition of the residual ozone on heterogeneous, environmentally friendly catalysts. In humid environments and under certain temperatures and gas flow rates this subject has not yet been entirely understood.

### 4.1. Stratospheric ozone

**Figure 4:**
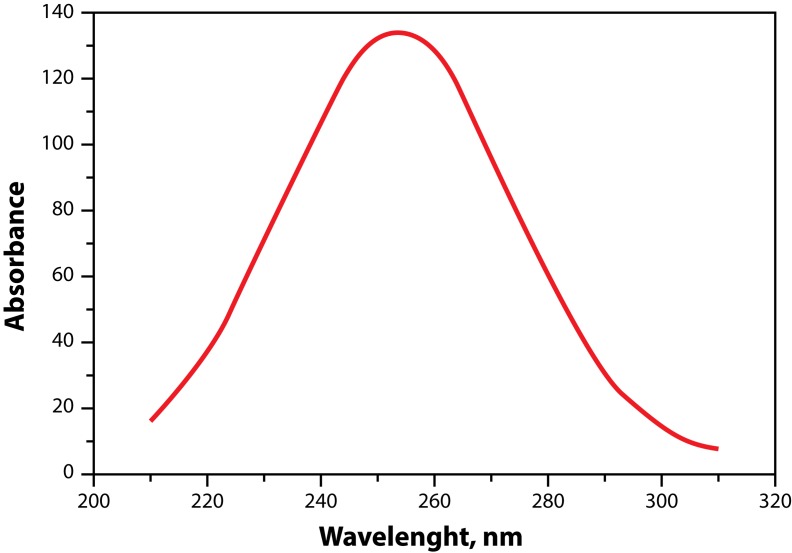
UV-absorption spectrum of O_3_ with maximum at 254 nm.

The importance of the stratospheric ozone is determined by its optical properties – its ability to absorb the UV solar radiation with a wavelength of 220 to 300 nm. The absorption spectrum of ozone in the ultraviolet and visible region is given in Figure 4.

The main absorption band in the name of Hartley is in the range of 200–300 nm with a maximum at the wavelength of 254 nm. The stratospheric ozone layer has a thickness of 3 mm and its ability to absorb UV rays protects the earth's surface of biologically active solar radiation which destroys the most important biological components, proteins and nucleic acids.

The fundamentals of the photochemical theory of stratospheric ozone were given by the English chemist Chapman (1930a, b), according to whom the photochemical decomposition of ozone occurs by the following reactions:

10 $${\text{O}}_{3}+\text{O}\to {\text{O}}_{2}+{\text{O}}_{2}$$

11 O3+hv→O2+O(P3)

12 O3+hv→O2+O(D1)

The proposed mechanism leads to formation of oxygen atoms in ground and excited state. This cycle of decomposition is called the cycle of “residual oxygen”. The photodissociation of ozone (11, 12) takes place under the action of solar radiation with a wavelength of less than 1134 nm. Decomposition of ozone following these reactions (11, 12) can be observed at any altitude near to ground level. The photochemical synthesis of ozone in the stratosphere requires its degradation in reaction (10). Following this reaction, 20% of the stratospheric ozone was found to decompose (Johnston, 1975). The effect of the ozone layer on climate changes is related to the absorption of radiation which occurs not only in the UV-region but also in the IR-region of the spectrum. By absorbing IR rays from the Earth's surface, ozone enhances the greenhouse effect in the atmosphere (Watson *et al.*, 1986; Gerchenson *et al.*, 1990). The connection of this problem with the climate is extremely complicated due to the variety of physical and chemical factors that influence the amount of atmospheric ozone (Johnston, 1975).

The destruction of ozone in the atmosphere is linked to catalytic cycles of the type:

$$\begin{array}{lll} \text{X}+{\text{O}}_{\text{3}}\to \text{XO}+{\text{O}}_{\text{2}} & & \text{X}+{\text{O}}_{\text{3}}\to \text{XO}+{\text{O}}_{\text{2}}\\ \underset{\_\_\_\_\_\_\_\_\_\_\_\_\_\_\_\_\_\_\_\_\_}{\text{XO}+\text{O}\to \text{X}+{\text{O}}_{\text{2}}} & & \underset{\_\_\_\_\_\_\_\_\_\_\_\_\_\_\_\_\_\_\_\_\_\_\_}{\text{XO}+{\text{O}}_{\text{3}}\to \text{X}+2{\text{O}}_{\text{2}}}\\ {\text{O}}_{\text{3}}+\text{O}\to {\text{O}}_{\text{2}}+{\text{O}}_{\text{2}} & & {\text{2O}}_{\text{3}}\to 3{\text{O}}_{\text{2}} \end{array}$$

where: X is OH, NO or Cl, formed by dissociation of freons in the atmosphere (Crutzen & Smalcel, 1983).

The stratospheric cycles of ozone decomposition have been discussed in several works but fundamental contributions have been made particularly by Johnston (1975) and Farmen *et al.* (1985). The effective action of the molecules-catalysts in the cycles of ozone decomposition is determined by their concentrations in the atmosphere, which depends on the rates of regeneration and exit from the respective cycle (Solomon *et al.*, 1986). The ratio between the rate of decomposition of residual oxygen and the rate of catalyst outgoing from the cycle determines the length of the chain reaction and corresponds to the number of O_3_ molecules destructed by one catalytic molecule. The number of stages of ozone decomposition in a single catalytic center can reach 106 (Rakovsky *et al.*, 2007). The reduction of the ozone amount over Antarctica, *i.e.* the thinning of the ozone layer, is mainly due to the action of the chlorine cycle (Vupputuri, 1988; Kondratev, 1989; 1990). During this catalytic cycle, the presence of one chlorine atom in the stratosphere can cause the decomposition of 100 000 ozone molecules.

### 4.2. Catalysts for decomposition of ozone

The use of ozone for industrial aims is related to the application of effective catalysts for its decomposition, since as already mentioned, the release of ozone in the atmosphere near ground level is dangerous and contaminates the air (Rakovsky *et al.*, 2007; Heisig *et al.*, 1997; Rakitskaya *et al.*, 2001). Ozone has a great number of advantages as an oxidizing agent and in that capacity it has been used in different scientific investigations concerned with neutralization of organic contaminants (Skoumal *et al.*, 2006; Bianchi *et al.*, 2006), as in the presence of a catalyst the ozonation efficiency increases (Ma *et al.*, 2004; Zhao *et al.*, 2008). Due to its antibacterial and antiviral properties, ozone is one of most used agents in water treatment (Von Gunten, 2003). This fact results in a considerable interest of researchers to study homogeneous and heterogeneous reactions of ozone decomposition, as well as the participation of ozone in multiple oxidation processes.

#### 4.2.1. Ozone decomposition on the surface of metals and metal oxides

The first studies on the catalytic decomposition of gaseous ozone (Monhot, 1907) showed that the degradation of ozone is accelerated in the presence of Pt, Pd, Ru, Cu, W, *etc.* The catalytic activity of metals in the decomposition of ozone was studied by Kashtanov *et al.* (1936) highlighting that silver (Ag) showed a higher catalytic activity compared to copper (Cu), palladium (Pd), and tin (Sn). From earlier works on the decomposition of ozone, the study of Schwab (Schwab & Hartmann, 1964) should be noted. They investigated catalysts based on metals from the I–IV group and their oxides in different oxidation levels and they found that the catalytic activity of these oxides increased with the increase in the oxidation state of the metal. Below the relationship is presented which exists between the catalytic activity degree of a number of elements and their oxides in the reaction of catalytic decomposition of ozone:

$$\text{Cu}<\text{C}{\text{u}}_{2}\text{O}<\text{CuO};\;\text{Ag}<\text{A}{\text{g}}_{2}\text{O}<\text{AgO};\;\text{Ni}<\text{N}{\text{i}}_{2}{\text{O}}_{3};\text{Fe}<\text{F}{\text{e}}_{2}{\text{O}}_{3};<\text{Au}<\text{A}{\text{u}}_{2}{\text{O}}_{3};\text{Pt}<\text{colloidal Pt}$$

These results can be explained by the higher activity of the ions of the respective elements relative to the activity of the elements themselves, as well as the importance of ionic charge in the catalytic reaction. Further early studies were presented by Emelyanova *et al.* (Emelyanova *et al.*, 1964; Emelyanova *et al.*, 1965; Emelyanova & Strakhov, 1968) discussing catalytic decomposition of gaseous ozone at temperatures from –80 °C to +80 °C and an ozone concentration of 8.8 vol.%. As catalysts elements of the platinum group were used: Pt, colloidal Pt, Pd, Ir and colloidal Ru, NiO, and Ni_2_O_3_. Experiments were carried out in a tube reactor at a gas velocity of 5 L.h^−1^. The studies indicated an identical activity of the nickel oxide and colloidal platinum at temperatures from 20 °C to 80 °C. At –80 °C, the activity of the nickel oxide fell to zero, while the platinum remained active. This is related to the low activation energy of decomposition of O_3_ on the metal surface (∼1 kcal.mol^−1^). Further it was found that the process of decomposition of ozone in the presence of colloidal Rh and Ir at +3 °C and +20 °C slowed down after four hours, and the catalysts lost activity. Sudak and Volfson's silver-manganese catalyst (Sudak & Volfson, 1983) for the decomposition of ozone was used at low temperatures in different gas mixtures. Commercial reactors operating with this catalyst have a long life of performance and maintain a constant catalytic activity. The preparation of other high performance metal catalysts for the decomposition of ozone, including the development of special technology for synthesis, determination of the chemical composition, experimental conditions and catalytic activity were presented in a number of studies by Japanese authors published in the patent literature (Hata *et al.*, 1988; Tchihara, 1988; Kobayashi *et al.*, 1988; Terui *et al.*, 1990; Terui *et al.*, 1991; Oohachi *et al.*, 1993; Kobayashi & Mitsui, 1988; Kuwabara & Fujita, 1991; Hata *et al.*, 1987; Terui *et al.*, 1990; Yoshimoto *et al.*, 1990). The main metals used were Pt, Pd, Rh and Ce, as well as metals and metal oxides of Mn, Co, Fe, Ni, Zn, Ag and Cu. The high price of precious metals stimulates the use of metal oxide catalyst supporters with a highly specific surface such as γ-Al_2_O_3_, SiO_2_, TiO_2_, ZrO_2_ and charcoal. Hata *et al.* (1988) synthesized a catalyst containing 2% Pt as active component on a supporter composed of a mixture of 5% SiO_2_ and 95% γ-Al_2_O_3_ having a specific surface area of 120 m^2^.g^−1^. They impregnated chloroplatinic acid onto the carrier at 80 °C, then the sample was dried successively at 120 °C and 400 °C and atmospheric pressure. At an operating temperature of 20 °C, the gas velocity of 20 000 h^−1^ and 10 ppm initial concentration of ozone in the fed gas, this catalyst shows 95% catalytic activity. Another catalyst comprising TiO_2_, SiO_2_ and Pt (Kobayashi *et al.*, 1988) degrades 94, 97 and 99% of the ozone in the air stream at temperatures of 20, 50 and 100 °C respectively. Therui *et al.* (1991) deposited the metals Mn, Co, Fe, Ni, Zn, Ag or their oxides in the weight ratio with regard to the carrier in the range of 0 to 60%, as well as Pt, Pd and Rh of the 0 to 10 wt %, and also mixed oxides such as TiO_2_-SiO_2_; TiO_2_-ZrO_2_ and TiO_2_-SiO_2_-ZrO_2_, supported on colloidal polyurethane with 400 m^2^.g^−1^ specific surface. The catalysts were placed in tube reactors and their catalytic activity, measured in the decomposition of ozone at a concentration of 0.2 ppm, was 99%. In published patents, various precursors and methods for synthesis were used for catalysts with identical chemical composition. For example, TiO_2_ and MnO_2_ (Kobayashi & Mitsui, 1988) were obtained from aqueous solutions of H_2_SO_4_ + TiOSO_4_ and Mn (NO_3_).6 H_2_O, while in another case (Kuwabara & Fujita, 1991), TiO_2_ was purchased from the manufacturer, and the MnO_2_ was prepared by precipitation of aqueous solutions of MnSO_4_ and NH_3_ in an atmosphere of oxygen followed by calcination. The application of the proposed system conditions makes it possible to assess the effectiveness of the catalysts and may be used for development of new catalytic systems. Many of the catalysts operate at ambient temperatures (293–323 K), high space velocities (>20 000 h^−1^), and exhibit high catalytic activity (conversion 95%). In recent years, more and more researchers have focused on the development, study and application of catalysts for decomposition of ozone based on supported or native metal oxides, with regard to the already mentioned fact – the high price of metals of the platinum group. In recent years, more and more researchers have focused on the development, study and application of catalysts for decomposition of ozone based on supported or unsupported metal oxides, due to the already mentioned fact – the high price of metals of the platinum group. In this respect, the most widely used are the oxides of Mn, Co, Cu, Fe, Ni, Si, Ti, Zr, Ag and Al (Oyama, 2000; Einaga & Futamura, 2004; Tong *et al.*, 2003; Konova *et al.*, 2006; Stoyanova *et al.*, 2006; Popovich *et al.*, 1985; Popovich, 1988; Radhakrishnan *et al.*, 2001; Zavadskii *et al.*, 2002). It has been found that the oxides of transition metals were found to exhibit the highest catalytic activity in the decomposition of ozone (Imamura *et al.*, 1991), particularly the catalysts based on manganese oxide (Dhandapani & Oyama, 1997). Manganese oxide is used as a catalyst for various chemical reactions including the decomposition of nitrous oxide (Lo Jacono & Schiavello, 1976; Yamashita & Vannice, 1996; Ma *et al.*, 1995) and isopropanol (Ma *et al.*, 1995; Ma *et al.*, 1995), oxidation of methanol (Baltanas *et al.*, 1986), ethanol (Li & Oyama, 1996), benzene (Naydenov & Mehandjiev, 1993; Einaga & Ogata, 2009), CO (Ma *et al.*, 1995; Ma *et al.*, 1995; Boreskov, 1965) and propane (Baldi *et al.*, 1998) as well as for reduction of nitric oxide (Kapteijn *et al.*, 1994) and nitrobenzene (Maltha *et al.*, 1994). In terms of technology for the control of air pollution, manganese oxides are used both in the decomposition of residual ozone and the degradation of volatile organic compounds (Hunter & Oyama, 2000; Subrahmanyam *et al.*, 2007). Oxides of Mn, Co, Ni, Cr, Ag, Cu, Ce, Fe, V and Mo supported on γ-Al2O3 on cordierite foam (60 pores per cm and geometry 5.1 × 5.1 × 1.3 cm) have been prepared and tested in the reaction of decomposition of ozone (Dhandapani & Oyama, 1997). Experiments were carried out at a temperature of 313 K, a linear velocity of 0.7 m.s^−1^, inlet ozone concentration of 2 ppm, a relative humidity of 40% and a total gas flow rate of 1 800 cm^3^.s^−1^. The catalyst activity was found to decrease over time and the measurements were made only when the speed of decomposition was stabilized. After comparison of the conversion degree of ozone, conclusions on their catalytic activity were drawn and they were arranged as follows: MnO_2_ (42%)> Co_3_O_4_ (39%)> NiO (35%)> Fe_2_O_3_ (24%)> Ag_2_O (21%)> Cr_2_O_3_ (18%)> CeO_2_ (11%)> MgO (8%)> V_2_O_5_ (8%)> CuO (5%)> MoO_3_ (4%). The high dispersibility of the material was confirmed by X-ray diffraction analysis. TPR profile of supported and unsupported MnO_2_ was done. It was found that the reduction temperature of the supported MnO_2_, Co_3_O_4_, NiO, MoO_3_, V_2_O_5_ and Fe_2_O_3_ was in the range of 611–735 K, while that of CeO_2_ was above 1 000 K. Of all the examined metal oxide samples, MnO_2_ had the lowest reduction temperature and exhibited a higher reduction ability in comparison with the others. On the basis of experiments, the mechanism of decomposition of ozone on the catalytic surface was proposed, including the formation of intermediate ionic particles possessing either superoxide or peroxide futures:

13 $${\text{O}}_{3}+2^{*}\to {}^{*}{\text{O}}_{2}+{\text{O}}^{*}$$

14 $${}^{*}{\text{O}}_{2}{+}^{*}\to 2{\text{O}}^{*}$$

15 $$2{\text{O}}^{*}\to {\text{O}}_{2}+2^{*}$$

where * denotes active center.

A distinguishing peculiarity of the proposed scheme is the assumption that the key intermediate particle *O_2_ is not desorbed immediately. This can happen if it has a partial ionic character (O^2–^, ${\text{O}}_{2}^{2-}$). The catalytic activity of iron oxide Fe_2_O_3_ in decomposition of ozone was studied also in earlier work of Rubashov (Rubashov *et al.*, 1972; Rubashov & Strahov, 1973). Fe_2_O_3_ was found to show a catalytic activity only when used in the form of particles with small diameter, while the catalyst consisting of aggregated particles was not efficient. Furthermore, the stability of the catalyst was not high and its poisoning was due to the formation of oxygen directly associated with the surface. Reactivation of the catalyst was accomplished by subjecting it to vacuum thermal treatment to remove oxygen. The same catalyst was also studied in terms of the fluidized bed, wherein the rate constant for the catalytic reaction is higher than in the normal gas flow. By determining the constant of the decomposition of ozone on the surface of Fe_2_O_3_ at various temperatures, the activation energy of the process: E=(12.5±1) kcal.mol^−1^ can be calculated. This value differs from that calculated by Schwab (Schwab & Hartmann, 1964) which is of the order of 2–3 kcal.mol^−1^. The difference can be explained by the regimen in which the process is carried out: kinetic, in the first case, and diffusion in the second. Ellis & Tomets (1972) investigated the activity of 35 different materials with respect to decomposition of ozone and nickel oxide and charcoal turned out to be the best catalysts. Measurements were carried out at room temperature, gas velocities between 250–2 000 L.h^−1^, atmospheric pressure and ozone concentrations (1.1–25) × 1012 molecules per cm^3^. The measured rate of decomposition of ozone corresponds to a first order reaction. The authors ignoring the decomposition of ozone in the bulk proposed a homogeneous decomposition on the surface:

17 $${\text{O}}_{3}+\text{O}\to 2{\text{O}}_{2}$$

In this case “M” is an active center on the surface which reduces the energy of activation of the reaction and accelerates decomposition. Assuming that the portion of the surface area occupied by ozone θ is proportional to the partial pressure of ozone in the volume, the kinetic region of ozone leaving from the gas phase is equal to:

18 $$-\frac{\mathrm{dp}o_{3}}{\mathrm{dt}}=k\theta =k^{\prime }po_{3}$$

It is obvious that a first order of reaction with respect to the decomposition of ozone has been established, which has been confirmed experimentally. The decomposition of ozone in gradient-free reactor is considered in the work (Houzellot & Villermaux, 1976). As a catalyst a thin film of nickel oxide coated on the walls of the reactor was used. Turbulence of gas flow in such a type of reactor would avoid the physical transfer and provide conditions for studying the kinetics only on the catalytic surface. In this case, the turbulent diffusion is superior to the rate of reaction and the process is conducted in the kinetic regimen. The rate constant in the range of 10–40 °C is calculated by the expression:

19 $$\text{k}=10^{2.54}p^{-0.5}\exp(-1250/\text{T})\text{cm}/\text{s}$$

where *p* is in atm.

Experiments made with different pressures of the oxygen and ozone-helium mixtures showed that the rate constant changes depend on the total pressure and not on the partial pressure of ozone. Hence the authors have concluded that the catalyst operates in inner diffusion mode, *i.e.* the reaction rate is limited by molecular diffusion in the pores of the catalyst. These and other assumptions made it possible to explain the resulting experimental dependence of the rate constant on the total pressure of the mixture in the gas phase. The kinetics of decomposition of ozone in gradient-free reactor was investigated in the work (Tarunin *et al.*, 1981). It measured activities of γ-Al_2_O_3_, hopkalite (MnO_2_, CuO, bentonite), silver-manganese oxide, alumina-palladium and alumina-platinum catalysts. The experiments were conducted at 60 °C and initial ozone concentration of 2.7×10^−5^ mol.L^−1^. The most active of the catalysts tested turned out to be silver-manganese oxide and hopkalite. The kinetics of decomposition of ozone has been determined to be of first order. The rate constants and energies of activation were also defined, the latter fluctuating in the range of 6–10 kcal.mol^−1^. Of interest are conclusions of authors on the active surface of the catalyst. The activation energy was found not to depend on the dispersibility, but influencing the catalytic activity. On the other hand, dispersibility did not increase the inner surface of the porous material. Hence it has been concluded that the decomposition of ozone occurs in the outer kinetic region, *i.e.* the heterogeneous reaction is limited mainly by the outer surface of the catalyst. During the study of the decomposition of ozone in the presence of nickel oxide (Rakovsky *et al.*, 1979) it was established that the reaction was of first order. It has been proposed that the limiting stage is the adsorption of ozone to the catalytic surface. The EPR spectra revealed the presence of ozonide ion radical O^3–^ (Che & Tench, 1982; Tench & Lawson, 1970). The formation of O^3–^ is explained by the process of electron transfer from the catalyst to the ozone, so that the surface Ni^2+^ ions are oxidized to Ni^3+^. Further studies of the authors showed that in this way approximately 5% of the total reaction took place. The remaining amount of the ozone was decomposed by molecular mechanism:

20
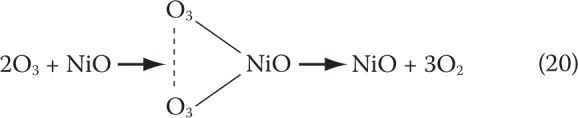



In some cases, the ozone does not remain in the molecular state and dissociates to atomic or diatomic oxygen species. Ozonide particles on catalytic surface are generated by the reaction:

21 $${\text{O}}^{-}+{\text{O}}_{2}\to {\text{O}}^{3-}$$

**Figure 5:**
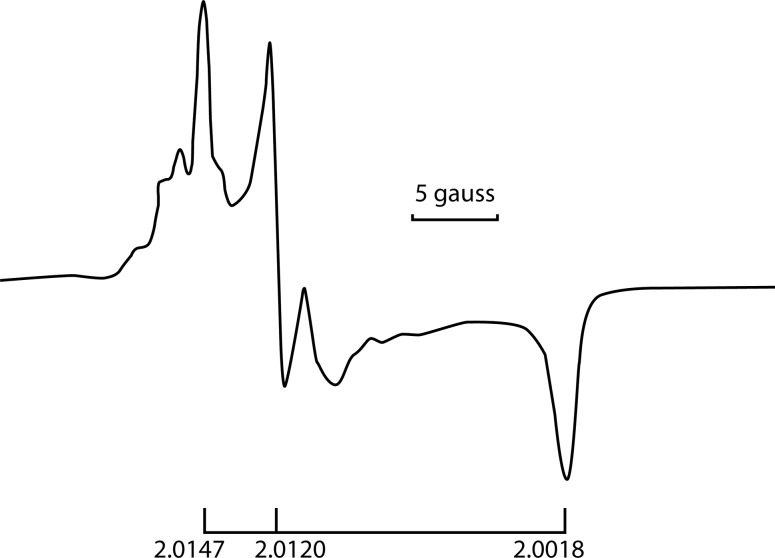
EPR spectrum of O^3–^ at a temperature 77 K.

Figure 5 shows the characteristic bands of the EPR spectrum at g_1_ = 2.0147, g_2_ = 2.0120 and g_3_ = 2.0018, due to the formation of the ion-radical O^3–^.

Martinov *et al.* (1994) studied the influence of the CuO, CoO and NiO on the process of ozone decomposition. The catalysts were prepared using aqueous solutions of the respective nitrates and were supported on the walls of the tubular reactor (l=25 cm, D=1.6 cm). The reactor was heated to 370 °C for 5 hours, after which each of the catalysts was treated with ozone at a concentration of 0.5 vol. % for 6 hours at a gas velocity of 100 L.h^−1^. After solving the diffusion-kinetic equation of the ozone decomposition reaction, the diffusion and the kinetic constants can be defined as well as the coefficients of ozone decomposition. These characterize the decomposition rate of ozone molecules on the catalyst surface toward the rate of the hits of molecules with the surface. The results of these calculations for different samples are presented in Table 4, wherein ω is space velocity, γ-coefficient of ozone decomposition and k_exp_ k_kin_ are respectively experimental and theoretical rate constants.

**Table 4:** Kinetic parameters of the reaction of ozone decomposition on some oxides.

Catalyst	w, l/h	k_exp_, s^−1^	g.10^−5^	k_kin_, s^−1^
CuO	20	1.0	4.8	2.8
CoO	100	0.9	4.1	2.4
CoO	200	1.3	6.3	3.6
NiO	20	0.9	4.4	2.6
NiO	100	1.0	4.8	2.8
NiO	200	1.7	8.1	4.4

It is evident that the activity of the catalysts increases at ω=200 L.h^−1^. This is explained by the flow of catalytic reaction between kinetic and diffusion regions. Furthermore, the values of the rate constants of ozone decomposition in kinetic mode are almost 3 times higher than the experimental ones.

Radhakrishnan *et al.* (2001) used manganese oxide catalysts supported on Al_2_O_3_, ZrO_2_, TiO_2_ and SiO_2_ for studying the support influence on the kinetics of decomposition of ozone. By using different physical methods of analysis, such as “*in situ*” laser Raman spectroscopy, temperature-programed desorption of oxygen and measuring of specific surface area (BET), the manganese oxide was found to be highly dispersed on the support surface. The Raman spectra of the supported catalysts revealed the presence of Mn-O bands as the result of well dispersed manganese oxide particles on the Al_2_O_3_ and SiO_2_ supported catalysts. During the process of ozone decomposition on the catalytic surface, a signal from adsorbed particles appears at Raman spectra in the region of 876–880 cm^−1^. These particles were identified as oxygen particles from peroxide type (${\text{O}}_{2}^{2-}$), which disappeared upon catalyst heating to 500 K. Using temperature-programed desorption of oxygen, the number of active manganese centers on the catalyst surface is calculated. After integration of the TPD peaks area corresponding to desorption of adsorbed oxygen, besides the density of active sites also the corresponding dispersion values of the catalysts are identified. These results, as the calculated specific surface areas of the catalytic samples, are shown in Table 5. The catalysts were tested in reaction of ozone decomposition to determine their activity, and it was found that the rate of decomposition increased with increasing the ozone partial pressure and temperature. The kinetic parameters of the reaction were calculated. The activation energy was in the range of 3–15 kJ.mol^−1^, depending on the catalyst sample, as it is lower (3 kJ.mol^−1^) in the case of ozone decomposition on MnO_x_/Al_2_O_3_. This may be related to the structure of this catalyst which was the only one of the samples tested with a mononuclear manganese center coordinated by five oxygen atoms, as demonstrated on using absorption fine-structure X-ray spectroscopy. The same method revealed that the other three supported catalysts possessed multi-core active manganese centers surrounded as well by five oxygen atoms.

**Table 5:** Densities of active centers and specific surfaces.

Catalyst	S_g_, m^2^.g^−1^	density, mmol.g^−1^ (O_2_/O_3_ TPD)	dispersion,%
MnO_x_/Al_2_O_3_	92	40	12
MnO_x_/ZrO_2_	45	163	47
MnO_x_/TiO_2_	47	31	9
MnO_x_/SiO_2_	88	13	4

A mechanism of ozone decomposition on MnO_x_/Al_2_O_3_ catalyst has been proposed:

22 $${\text{O}}_{3}+\text{M}{\text{n}}^{n+}\to {\text{O}}^{2-}+\text{M}{\text{n}}^{(n+2)+}+{\text{O}}_{2}$$

23 $${\text{O}}_{3}+{\text{O}}^{2-}+\text{M}{\text{n}}^{(n+2)+}\to {\text{O}}_{2}^{2-}+\text{M}{\text{n}}^{(n+2)+}+{\text{O}}_{2}$$

24 $${\text{O}}_{2}^{2-}+\text{M}{\text{n}}^{(n+2)+}\to \text{M}{\text{n}}^{n+}+{\text{O}}_{2}$$

The presented mechanism consists of electronic transfer from the manganese center to ozone, followed by reduction of manganese by desorption of peroxide particles to form oxygen (${\text{O}}_{2}^{2-}$ → O_2_ + 2e^−^).

A study of MnO_x_/Al_2_O_3_ with absorption fine-structure X-ray spectroscopy is presented also in publication (Einaga *et al.*, 2005). The aim of the work was to receive important information on the catalytic properties of the supported manganese oxides in oxidation reactions using ozone. The structural changes in the manganese oxide supported on alumina were detected in the process of catalytic ozone decomposition at room temperature. It was found that during the ozone decomposition in the presence of water vapor, the manganese was oxidized to a higher oxidation state. At the same time the water molecule combined to manganese active center due to cleavage of the Mn-O-Al bond. The catalyst was completely regenerated after calcination in oxygen at 723 K.

The paper (Martinov *et al.*, 1999) studied the influence of nickel oxide addition on the activity of cement containing catalyst for ozone decomposition. The activity of the samples was measured by calculating the rate of decomposition γ which shows the degree of active interactions (leading to decomposition) of ozone molecules with catalytic surface. According to Lunin *et al.* (1998), the expression for γ is:

25 $$\gamma =\frac{4\omega .\ln\left({\text{C}}_{0}/\text{C}\right)}{{\text{V}}_{\text{t}}.\text{S}}$$

where: V_t_ - specific heat velocity of ozone molecules; S - geometric surface of the catalyst; ω - space velocity of the gas stream; C_0_ and C - inlet and outlet ozone concentrations.

Addition of nickel oxide to the catalyst composition improved its catalytic properties. Upon decomposition of wet ozone, a decrease in the activity of all samples tested was found, as the calculated values for γ were 2–3 times lower compared with the value obtained after decomposition of dry ozone. On the basis of the values measured for energy of activation (E_a_ = 5.9±0.3 kJ.mol^−1^) of ozone decomposition in the region of high temperatures (300–400 K), and the corresponding values of E_a_ (15.2±0.4 kJ.mol^−1^) in the region of low temperatures, the conclusion was drawn that in the first case the process went to the outer diffusion region, whereas in the second into the inner diffusion region. The factor of diffusion suspension or the accessible part of the surface is estimated to elucidate the role of the internal catalytic surface. This permitted the calculation that at room temperature the molecules of ozone enter into the pores of the catalyst at a distance not greater than ∼10^−4^ cm.

Lin *et al.* [97] studied the activity of a series of oxide supports and supported metal catalysts with respect to decomposition of ozone in water. Tested in reaction conditions, activated carbon showed a relatively high activity, while the zeolite support (HY and modernite), Al_2_O_3_, SiO_2_, SiO_2_.Al_2_O_3_ and TiO_2_ showed zero or negligible activity. Of all the supported metal catalysts submitted to ozone dissolved in water, the noble metals had the highest activity in ozone decomposition, except gold. The metals are deposited on four types of supports (Al_2_O_3_, SiO_2_, SiO_2_.Al_2_O_3_ and TiO_2_). The highest activity was measured for the catalysts deposited on silica. Of all the samples tested, the catalyst containing 3% Pd/SiO_2_ was found to be the most effective in the reaction of ozone decomposition. A comparison of some indicators for Pd catalysts deposited on different supports is presented in Table 6.

**Table 6:** Comparison of the specific surface areas, the size of metal particles and the average rates of ozone decomposition in water over palladium-containing catalysts.

Catalyst	Specific surface area (m^2^.g^−1^)	Size of metal particle (Å)	Average rate (mg_(O_3_)_ min^−1^.g^−1^_(cat.)_)
Pd/SiO_2_	206	90	0.77
Pd/SiO_2_.Al_2_O_3_	221	70	0.54
Pd/Al_2_O_3_	139	75	0.39
Pd/TiO_2_	34	109	0.35

It was determined as the first order of reaction of ozone decomposition on 3% Pd/SiO_2_ regarding the concentration of ozone. In the presence of the same catalyst, the calculated activation energy was about 3 kcal.mol^−1^ and it is assumed that the reaction proceeds in the diffusion region. The proposed mechanism of catalytic decomposition of ozone in water is similar to the mechanism of decomposition of gaseous ozone. Table 7 shows two possible reaction pathways of ozone decomposition on metals or oxides, depending on the fact whether oxygen is adsorbed on the catalytic surface.

**Table 7:** Possible mechanisms of catalytic ozone decomposition in water (Lin *et al.*, 2002).

Case of O_2_ not adsorbed on metal	Case of O_2_ adsorbed on metal
O_3_ → O_3(a)_	O_3_ → O_3(a)_
O_3(a)_ → O_(a)_ + O_2_	O_3(a)_ → O_(a)_ + O_2(a)_
O_(a)_ + O_3_ → 2O_2_	O_(a)_ + O_3_ → O_2_ + O_2(a)_ O_2(a)_ → O_2_

The literature provides also data for the study of ozone decomposition in water in the presence of aluminum (hydroxyl) oxide (Qi *et al.*, 2008). It has been suggested that the surface hydroxyl groups and the acid-basic properties of aluminum (hydroxyl) oxides play an important role in catalytic decomposition of ozone.

The environmental application of ozone in catalysis was demonstrated in article (Rosal *et al.*, 2008), devoted to the ozonation of naproxen and carbamazepine on titanium dioxide. The experiments were carried out in aqueous solution at T = 25 °C and in pH range of 3–7. The results indicated that naproxen and carbamazepine are completely destructed in the first few minutes of the reaction. The degree of mineralization during the non-catalytic reaction flow was measured to be about 50%, formed primarily in the first 10–20 minutes. The presence of the catalyst increased to more than 75% the degree of mineralization of the initial hydrocarbon. Furthermore, it was found that the catalyst increased the mineralization in both acid and neutral solution, as the best results were obtained at slightly acidic media. This effect may be related with a possible adsorption of intermediate reaction products on Lewis acidic catalytic sites. The titanium dioxide possibly catalyzes the ozone decomposition in acidic media, whereas in neutral solution the ozone destruction is inhibited. This precluded the flow of mechanism based on surface formation of hydroxyl radicals followed by their migration and complete reaction with the organic compounds. The variation of the quantity of total organic carbon is modeled as a function of the integral of the applied amount of ozone. On this basis, it is assumed that the reaction between organic compounds and ozone is of second order. The calculated pseudo homogeneous catalytic rate constants are 7.76 × 10^−3^±3.9 × 10^−4^ and 4.25 × 10^−3^±9.7 × 10^−4^ L.mmol^−1^.s^−1^ for naproxen and carbamazepine, respectively, at pH 5 and catalyst amount of 1 g.L^−1^. The products of ozonation are investigated with a specific ultraviolet absorption at 254 nm. The wide application of metal oxide catalysts in ozone decomposition necessitated the use of different instrumental methods for analysis. Based on the results of X-ray diffraction, X-ray photoelectron spectroscopy, EPR and TPD, it was found that during the destruction of ozone on silver catalyst supported on silica, the silver was oxidized to a complex mixture of Ag_2_O_3_ and AgO (Naydenov *et al.*, 2008). This investigation of catalytic ozone decomposition on Ag/SiO2 was carried out in the temperature range from –40 °C to +25 °C, it was determined first order of reaction, and the calculated activation energy was 65 kJ.mol^−1^. X-ray diffraction was also used to determine the phase composition of manganese oxide catalysts supported on γ-Al_2_O_3_ and SiO_2_ (Buciuman *et al.*, 1998). Moreover, the samples were characterized by Raman and IR spectroscopy. The supported catalysts were prepared by nitrate precursors using the impregnation method. In addition to the Raman spectral bands of β-MnO_2_ and α-Mn_2_O_3_ phases, other signals were also registered and attributed to isolated Mn^2+^ ions present in tetraedrical vacations on the support surface and in some epitaxial layers, respectively on γ-Mn_2_O_3_ and manganese silicate. The data from the IR spectra were not very useful due to the fact that the supporter band overlapped the bands of manganese particles formed on the surface and made it difficult to identify them.

Eynaga and Futamura (2005) carried out catalytic oxidation of cyclohexane with ozone on manganese oxide supported on aluminum oxide at the temperature of 295 K. It was performed *"in situ"* IR studies for taking information on the intermediates formed on the catalytic surface at the time of oxidation. The intermediates were partially oxidized alcohols, ketones, acid anhydrides and carboxylic acids. These compounds were subsequently decomposed by ozone. In the beginning, the activity of the catalyst gradually decreased, whereupon it reached a steady state with mole fractions of CO and CO_2_ of respective 90% and 10%. High resistant particles containing C=O, COO- and CH groups remained on the catalyst surface, causing slow deactivation of the catalyst. The C=O groups were decomposed at relatively low temperatures (<473 K), while the COO- and CH groups were dissociated at temperatures> 473 K.

The kinetics of gas-phase ozone decomposition was studied by heterogeneous interactions of ozone with aluminum thin films (Sullivan *et al.*, 2004). The ozone concentrations were monitored in real time using UV absorption spectroscopy at 254 nm. The films were prepared by dispersion of fine alumina powder in methanol, and their specific surface areas were determined by “in situ” adsorption of krypton at 77 K. The reactivity of alumina decreased with increasing ozone concentration. As a consequence of multiple exposures to ozone of one film, the number of active sites was greater than 1.4 × 1014 per cm^2^ surface or comparable with the total number of active sites. The coefficients of ozone decomposition were calculated (γ) depending on the initial concentration of ozone in the reaction cell, using the expression:

26 $$\gamma =\left(4\times k^{I}\times V\right)/\left(c\times SA\right)$$

where c is the average velocity of gas phase molecules of ozone, k^I^ is the observed initial rate constant of ozone decomposition from first order (for the first 10 s), SA is the total surface area of the aluminum film, and V is volume of the reactor. The results indicate that the coefficients of ozone decomposition on fresh films depend inversely on ozone concentration, ranging from 10^−6^ to ozone concentration of 10^14^ molecules per cm^3^ to 10^−5^ for 10^13^ molecules per cm^3^ ozone. The coefficients of ozone decomposition were found not to depend on the relative humidity of gas stream. The mechanism of the reaction of catalytic ozone decomposition based on detailed spectroscopic investigations of the catalytic surface was discussed (Li *et al.*, 1998; Li & Oyama, 1998), with evidence for the formation of peroxide particles on the surface:

27 $${\text{O}}_{3}+{}^{*}\to {\text{O}}_{2}+{\text{O}}^{*}$$

28 $${\text{O}}_{3}+{\text{O}}^{*}\to {\text{O}}_{2}^{*}+{\text{O}}_{2}$$

29 $${\text{O}}_{2}^{*}\to {\text{O}}_{2}+{}^{*}$$

It has been suggested that molecular oxygen could also be initiated by the reaction:

30 $${\text{O}}^{*}+{\text{O}}^{*}\to 2^{*}+{\text{O}}_{2}$$

The reactivity of the oxidized aluminum film can be partially restored after being placed for a certain period in a medium free of ozone, water vapor and carbon dioxide.

#### 4.2.2. Decomposition of ozone on the surface of the carbon fiber and inert materials

The use of carbon material in adsorption and catalysis is related to their structural properties and surface chemical groups. Their structural properties are determined by the specific surface area and porosity, while the chemical groups on the surface of the catalyst are composed mainly of oxygen-containing functional groups. Ozone reacts with various carbon materials such as activated carbon, carbon black, graphite, carbon fiber, *etc.* The character of these interactions depends both on the nature of the carbon surface and temperature (Atale *et al.*, 1995). It has been tested two. Two types of interactions were tested: complete oxidation leading to formation of gaseous carbon oxides and partial oxidation producing surface oxygen-containing functional groups (Atale *et al.*, 1995; Mori *et al.*, 1993). With increase of temperature, the ratio between these two processes is displaced in the direction of complete oxidation. The latter is accompanied by an oxidative destruction on the surface with formation of gaseous carbon oxides. In addition, on the surface of the carbon catalytic decomposition of ozone takes place. A number of publications (Kobayashi *et al.*, 1989; Rakitskaya *et al.*, 1994; Aktyacheva & Emelyanova, 1990; Valdes *et al.*, 2002) studied the physicochemical properties of activated carbon treated with ozone, as well as and the kinetics of the process related to the release of CO and CO_2_. Subrahmanyam *et al.* (2005) have examined the catalytic decomposition of ozone to molecular oxygen on active carbon in the form of granules and fibers at room temperature. The dynamics of the activity of the carbons was characterized by two distinct zones. The first one is the observed high activity with respect to the decomposition of ozone, which is mainly due to the chemical reaction of ozone with carbon. As a result of this interaction on the carbons, oxygen-containing surface groups are formed. Then a sharp drop of the conversion is registered and transition of the catalyst to a low active zone takes place. In this zone, the decomposition of the ozone to molecular oxygen proceeds in a catalytic way. The activities of the carbons in dry environment on the one hand and in the presence of water vapor and NO_x_ on the other were compared. The presence of water vapor reduced the catalytic activity, while the presence of the NO_x_ improved the activity due to the change in the carbon surface functional groups.

**Figure 6:**
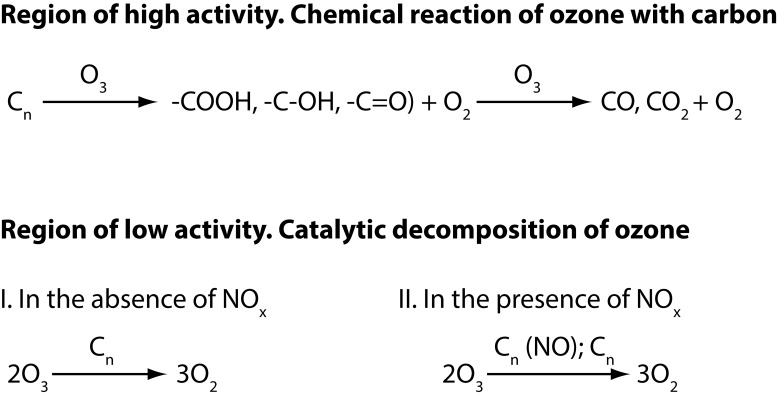
Simplified scheme of ozone decomposition on carbon.

They can be modified in two ways: boiling in dilute nitric acid or thermal treatment at 1 273 K in helium media. Positive results were obtained only in the first treatment. The decomposition of ozone with respect to the gasification of carbon with forming of CO_x_ proceeds with a selectivity of less than 25%. The catalysts were characterized by means of temperature-programed decomposition of surface functional groups, IR and X-ray photo-electron spectroscopy. The mechanism of decomposition of ozone on activated carbon has been proposed (Figure 6).

In the ozone decomposition process it is difficult to draw a line between relatively inert materials and heterogeneous catalysts. However the separation can be made on the basis of two evident signs: 1) the catalysts for ozone decomposition are specifically synthesized and 2) they have higher catalytic activity (higher values for the coefficient of decomposition γ) compared to the inert materials. In the literature there are investigations on the reaction of ozone destruction on the surface of silica (Tkalich *et al.*, 1984), glass (Olshina *et al.*, 1979), volcanic aerosols (Popovich *et al.*, 1987; Popovich, 1988) and ammonium hydrogen sulfate (Egorova *et al.*, 1988). The published values for the coefficient of ozone decomposition γ for quartz and glass are in the order of (1–2) × 10^−11^, and those for aerosols and ammonium sulphate–from 1.61 × 10^−6^ to 7.71 × 10^−8^. It is interesting to note the studies of ozone decomposition on the surface of Saharan dust (Hanisch & Crowley, 2002) as well as the application of slurry treated water as catalyst for ozone destruction in aqueous medium (Muruganadham *et al.*, 2007).

The heterogeneous reaction between O_3_ and authentic Saharan dust surfaces (Hanisch & Crowley, 2002) was investigated in a Knudsen reactor at ∼296 K. O_3_ was destroyed on the dust surface and O_2_ was formed with conversion efficiencies of 1.0 and 1.3 molecules O_2_ per O_3_ molecule destroyed for unheated and heated samples, respectively. No O_3_ desorbed from exposed dust samples, showing that the uptake was irreversible. The uptake coefficients for the irreversible destruction of O_3_ on (unheated) Saharan dust surfaces depended on the O_3_ concentration and varied between 4.8×10^−5^ and 2.2×10^−6^ for the steady-state uptake coefficient. At very high O_3_ concentrations the surface was deactivated, and O_3_ uptake ceased after a certain exposure period.

A new, effective and stable ecological catalyst based on slurry (Muruganadham *et al.*, 2007) was used in the process of ozone decomposition in water acidic medium. The catalyst was characterized by X-ray fluorescence, transmission electron microscopy, scanning electron microscopy and X-ray diffraction. The sludge is essentially composed of different metallic and non-metallic oxides. The effect of various experimental parameters was investigated, including catalyst amount, initial ozone concentration and application of different metal oxide catalysts. The decomposition of dissolved ozone is significantly increased with the enhancement of the initial ozone concentration and the increment of catalyst amount from 125 to 750 mg. The order of activity of the catalysts tested was as follows: ZnO≈sludge>TiO_2_>SiO_2_>Al_2_O_3_≈Fe_2_O_3_. Ozone did not affect the catalyst morphology and its composition and it is concluded that sludge is a promising catalyst for ozone decomposition in water.

On balance of the literature review it can be concluded that except metals of the platinum group, characterized by their high price, the metal oxide catalysts containing manganese oxide have the highest activity in decomposition of gaseous ozone and also in catalytic oxidation of pollutants. It is important to mention that unlike the inert materials, the oxide catalysts do not show strong dependence of catalytic activity on ozone concentration in gas phase. It should be emphasized that despite the great number of publications on the subject, the kinetics and the mechanism of ozone decomposition on the surface of heterogeneous metal oxide catalysts have not yet been cleared up sufficiently.
